# *Staphylococcal aureus* outbreaks in neonatal intensive care units: strategies, nuances, and lessons learned from the frontline

**DOI:** 10.1017/ash.2024.59

**Published:** 2024-05-02

**Authors:** Christopher M. Rooney, Rebecca Lancaster, Liz McKechnie, Kavita Sethi

**Affiliations:** 1 Department of Microbiology, Leeds Teaching Hospitals NHS Trust, Leeds, Yorkshire, England, UK; 2 Leeds Institute of Medical Research, University of Leeds, Yorkshire, England, UK; 3 Leeds Centre for Newborn Care, Leeds Children’s Hospital, Leeds, Yorkshire, England, UK

## Abstract

Methicillin-resistant *Staphylococcus aureus* (MRSA) infections in neonates can result in significant morbidity and mortality. However, comparatively to adults, neonatal MRSA data remains relatively scarce. Additionally, while evidence-driven practices for adults have seen considerable progress, neonatal infection prevention strategies remain poorly described. The Leeds Newborn Service adopted a series of infection prevention and control (IPC) measures following a rise in MRSA cases in 2008–2009. This narrative review presents IPC measures for neonatal MRSA and methicillin-sensitive *Staphylococcus aureus* (MSSA) infections and reflects upon local challenges and successes of these interventions. Our experience underscores the importance of an adaptive, evidence-based strategy, tailored to the neonatal population. Effectively addressing MRSA/MSSA requires continuous monitoring with sustained targeted interventions. Our key learning points highlight the intertwined difficulties of specific neonatal requirements and lack of definitive IPC guidance, suggesting a holistic approach is key for successful IPC outcomes in the neonatal intensive care unit setting.

## Introduction

Neonatal intensive care units (NICUs) are critical care environments where the prevention of microbial transmission is paramount. Among the array of potential pathogens, methicillin-resistant *Staphylococcus aureus* (MRSA) and methicillin-sensitive *Staphylococcus aureus* (MSSA) have emerged as significant threats. This narrative review evaluates infection prevention and control (IPC) strategies for MRSA and MSSA management, incorporating local experience and highlighting specific challenges and successes.

The acquisition of MRSA in NICUs can be attributed to various factors ranging from cross-contamination to prolonged antibiotic use.^1^ The first neonatal case of MRSA infection within a NICU was published in 1981, 20 years after the first MRSA case was described.^2^ *Staphylococcus aureus* has now become an increasingly problematic bacterial pathogen within NICUs worldwide. Preterm, very low birth weight, and critically ill neonates are particularly vulnerable to invasive *S. aureus* infections as they are immunologically immature and potentially undergo multiple invasive procedures.^1^ Controlling MRSA transmission in NICUs represents a significant challenge as many healthcare workers, parents/family members, and visitors are asymptomatically colonized and unknowingly serve as reservoirs for transmission.^1^ Hence, specific interventions targeting primary caregivers may be required to reduce transmission.^3^ The ability of *S. aureus* to survive for prolonged periods on environmental surfaces increases the difficulty in controlling transmission, especially within augmented care units.^4^

The impact of MRSA infection has been well documented in adult patients, with prior MRSA colonization representing a significant risk factor.^5^ In adults, MRSA infection is associated with a 2–3-fold increased risk of mortality, 2–4 days longer in-patient stay, and $6,000–$14,000 excess charges when compared with MSSA bacteremia or surgical site infections.^6^ This granularity of data is lacking for neonates. However, it has been reported that late-onset neonatal sepsis may prolong in-patient stay by 3 weeks and increase mortality from 7% to 18%.^7^ Demographic and epidemiological data on *S. aureus* neonatal septicemia in England is lacking. While the UK Health Security Agency’s Data Capture System contains *S. aureus* mandatory surveillance data, at present, the system does not stratify these data according to the neonatal population, but rather to children aged 0–1 years. Currently, local epidemiology informs hospital neonatal MRSA policy, with considerable variation in infection prevention practices, which are largely based on evidence from adult populations. MRSA surveillance has reduced horizontal spread within the United Kingdom in neonatal centers. However, no universal, specific recommendations exist to guide the surveillance and management of MRSA in the NICU.

## Current NICU practice and interventions adapted from adult ICUs

Current practices for preventing invasive disease due to MRSA in the NICU include a “seek and destroy” infection control program, whereby periodic surveillance cultures are obtained and colonized infants are decolonized and/or isolated.^8^ Decolonization procedures are unit based, gestational, and chronological age specific. A survey of UK NICUs demonstrated that 73.8% of MRSA-colonized neonates underwent routine decolonization.^9^ Furthermore, the choice of decolonization regimen varied, with more than 15 different regimens in use. Subsequent source isolation after successful decolonization also varied among the units.^9^

Universal decolonization in adult intensive care units (ICUs) has been found to be more effective than targeted decolonization or screening and isolation in reducing rates of MRSA clinical isolates and bloodstream infection from any pathogen. Huang et al used a pragmatic, cluster-randomized trial to compare 3 strategies for reducing MRSA infections in 74 adult ICUs, across 43 American hospitals.^10^ The strategies included MRSA screening and isolation and targeted decolonization (screening, isolation, and decolonization of MRSA carriers) and universal decolonization (no screening, but decolonization of all patients). The trial demonstrated universal decolonization reduced MRSA-positive clinical cultures by 37% and bloodstream infections from any pathogen by 44%, and adverse events were mild and related to chlorhexidine.^10^ It remains to be seen whether universal decolonization can obviate the need for all contact precautions for carriers of MRSA or other multidrug-resistant organisms.^10^

## Challenges with interventions in neonates

To reduce the morbidity and mortality associated with invasive MRSA infection, screening of infants in neonatal care facilities has become common practice. However, body site and frequency of screening are not universally agreed upon, and as previously mentioned, the prevalence of MRSA in each locality may necessitate different practices.^11–13^ The US Centers for Disease Control and Prevention (CDC) *S. aureus* NICU guideline, published in 2021, stipulates as a minimum, the anterior nares should be sampled in NICU patients, based on moderate evidence from two diagnostic studies.^14^ Recently updated National Institute of Clinical Excellence (NICE) guidelines on the management of MRSA have removed a recommendation specifically listing umbilicus and throat as preferential screening sites in neonates, citing a lack of evidence.^15^

Additionally, targeted MRSA decolonization strategies have limitations. First, despite weekly surveillance cultures, 42% of infections had no preceding positive MRSA screening swab, providing no opportunity to attempt decolonization.^8^ Second, the interval between colonization and infection in many neonates was short (median 5 days), suggesting a narrow window of opportunity for decolonization. Third, 38% of neonates who received decolonization treatment became recolonized during their NICU stay, and 16% developed an MRSA infection, so the efficacy of decolonization to eradicate MRSA colonization and prevent MRSA infections may be limited.^16,17^ Some authors have described the universal treatment of all neonates with mupirocin to successfully reduce MRSA infections in neonates.^18^ However, a universal approach in some settings has led to the emergence of mupirocin resistance, and the long-term impact of indiscriminately treating all neonates, including those not colonized with *S. aureus*, is unknown^19^ and may negatively affect the neonatal microbiome.^20^

The optimal decolonization agent or combination of agents remains an unresolved issue. As reported in the product specifications, the safety and efficacy of intranasal mupirocin are not established in patients aged less than 12 years. Additionally, in neonates and premature infants, systemic absorption occurs following intranasal administration, but it remains uncertain whether this absorption causes adverse health consequences in neonates. The application of a nasal ointment can be technically challenging in very low birth weight infants. Furthermore, certain agents, such as topical chlorhexidine, can cause adverse skin reactions, including chemical burns, especially in premature infants.^21^ A prospective randomized trial is needed to formally evaluate the efficacy and safety of mupirocin with or without chlorhexidine for eradicating MRSA colonization and preventing MRSA infections in neonates. This could be expanded to include primary caregivers and family.

Duffy et al examined 21,736 surveillance specimens from 3,784 admissions to a tertiary neonatal unit over an 8-year period.^22^ They demonstrated infants colonized with MRSA were smaller and of lower gestational age. Furthermore, infants initially positive on groin swabs tended to have greater gestational age and weight at birth compared with those initially positive on nose swabs or both nose and groin swabs.^22^ Duffy et al concluded weekly nasal swabs and swabbing of suspected infected sites could detect 85% of colonized infants, while groin swabs detected only a small number of larger, mature babies who were discharged before 2 weeks of age.^22^

## Description of the Leeds neonatal service

The Leeds Neonatal Service admits approximately 1,600 neonates annually. The service operates over 2 sites: the first functions as a Level 1 special care baby unit (20 cots) and the second as a Level 3 neonatal intensive care unit with surgery (34 cots). The latter provides care for both inborn and outborn extremely preterm infants and neonates requiring specialized surgical and cardiac care. Given this profile, these neonates are at an increased risk of developing *S. aureus* infections. Admission swabs from both outborn and inborn neonates have similar MRSA rates, as did the 2 neonatal units, and therefore for this study, the data from both units were analyzed together.

Nationally, *S. aureus* bacteremia exhibited a remarkable surge in MRSA strains during the 1990s, followed by a subsequent stabilization and decline between 2000 and 2007.^23^ Although MRSA infections are predominantly observed in older patients, data from 1990 to 2001 highlighted an increase in MRSA bacteremia among children, with the number of cases rising from less than 5 (constituting <1% of all *S. aureus* cases) in 1990 to over 70 in 2000 and 2001 (representing >10% of all *S. aureus* cases).^24^ It is suspected that neonates accounted for most of these cases, although the granularity of data is not captured.

In 2008–2009, the Leeds neonatal service recorded the highest number of MRSA bacteremia cases in the United Kingdom, with 8 instances, surpassing the total of 7 cases reported by 15 other neonatal units combined. The baseline rate for all hospital-onset MRSA for 2008–2009 in England was reported as 3.3 cases per 100,000 acute trust days [Mandatory surveillance data]. Leeds Teaching Hospitals Trust (LTHT) had a rate of 9.8 for the same time frame. However, it is important to highlight that LTHT is one of the largest acute trusts in England with an estimated 653,228 bed days compared with the average of 325,950, for the same period. Due to the associated morbidity of MRSA bacteremia, the neonatal service undertook a 4-week assessment period and systematic review of cases. The assessment included environmental audits, a revision of the junior medical staff induction procedures, ward housekeeping/cleaning inspections, daily hand hygiene audits, and an evaluation of antimicrobial prescription practices.

## Interventions undertaken in the Leeds neonatal service

**Figure 1.**
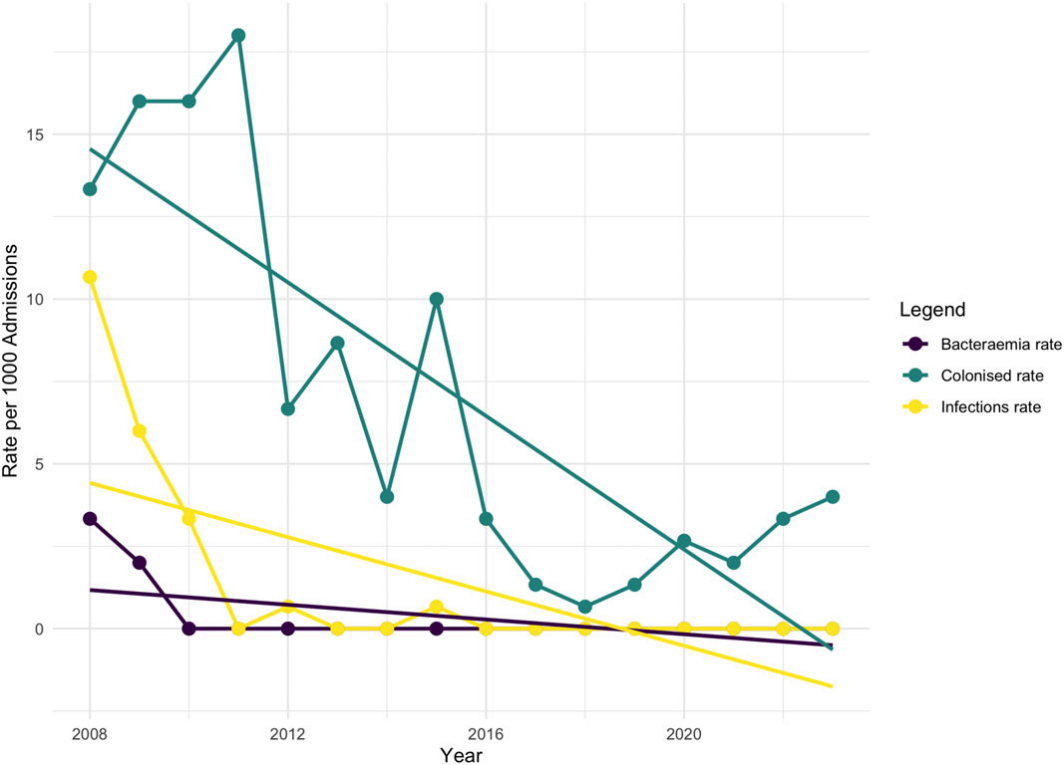
Methicillin-resistant *Staphylococcus aureus* (MRSA) isolation rates per 1,000 admissions in the Leeds neonatal service, from 2008 until 2023. Infection rate refers to skin and soft tissue infections only; bacteremia rate refers to the isolation of MRSA from blood.

**Figure 2.**
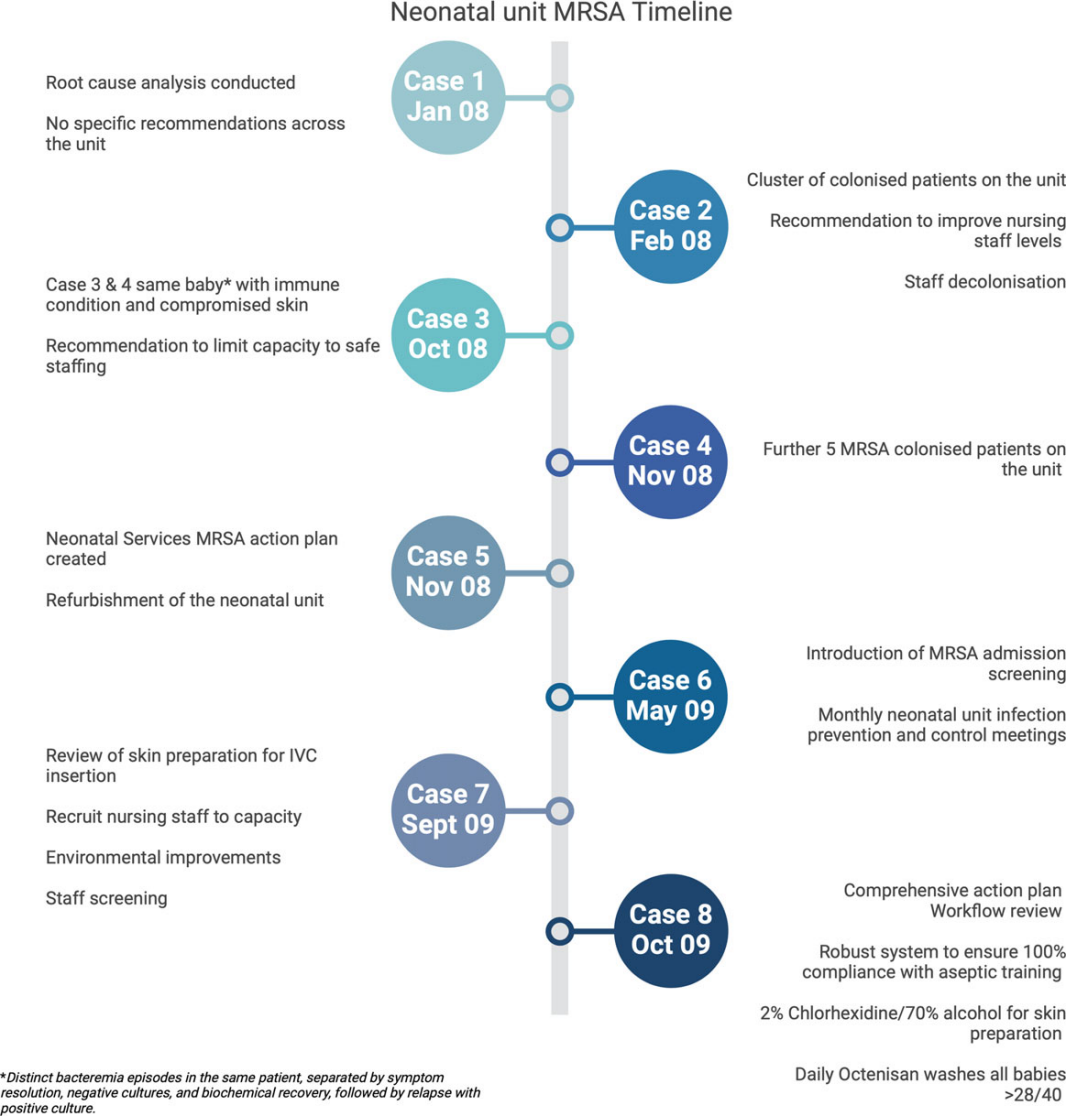
The Leeds neonatal service methicillin-resistant *Staphylococcus aureus* (MRSA) time line. Actions undertaken associated with each case are described. Each circle represents a month and year.

Figure 1 demonstrates the rising MRSA isolation from patients within the Leeds neonatal service. A root cause analysis (RCA) of bacteremia cases demonstrated that patients with Broviac or peripherally inserted central catheter lines, current MRSA colonization, previous abdominal surgery, and discharging skin lesions were at higher risk of MRSA bacteremia. Following RCA analysis, 3 domains were targeted for focused intervention including healthcare staff, the environment, and the patient. The interventions are detailed in Figure 2 and summarized in Table 1.

**Table 1.** A summary of key interventions to target methicillin-resistant *Staphylococcus aureus* (MRSA) bacteremia. The table summarizes the interventions undertaken, divided broadly into three categories

Category	Interventions
Staffing	Recruitment to improve the nurse-to-patient ratio, universal aseptic training and e-learning, staff engagement activities, nursing empowerment, MRSA screening and decolonization for staff, weekly interdisciplinary infection control meeting
Environment	Reassessment of cot spacing, de-cluttering and organization of the unit, improved access to PPE, comprehensive audit of ward cleaning practices
Patient	MRSA colonization awareness, weekly MRSA screening, daily Octenisan skin washes for neonates >28 weeks, use of 2% chlorhexidine for skin asepsis before invasive procedures, change of dressings, universal decolonization approach

Note. PPE, personal protective equipment.

Addressing understaffing was thought to improve adherence to infection control standards; therefore, recruitment was conducted to align the nurse-to-patient ratio with the British Association of Perinatal Medicine standards (BAPM).^25^ A system was implemented to achieve universal compliance with aseptic training and e-learning. The IPC strategy also included staff engagement activities and nursing empowerment, focusing on education to enable staff to challenge poor practices. Voluntary staff MRSA screening and decolonization was undertaken, offering onetime screening and decolonization for positives, with no known failures in staff decolonization.

Additionally, a weekly interdisciplinary infection control meeting was initiated to streamline communication and improve practices. Considering the environmental aspect, ward modifications included a reassessment of cot spacing, de-cluttering, and organization of the unit for enhanced functionality, improved access to personal protective equipment (PPE), and a comprehensive audit of ward cleaning practices.

A series of patient-focused infection control interventions were introduced, including weekly MRSA screening (axilla, groin, umbilicus, and nose), daily antiseptic skin washes (Octenisan) of neonates above 28 weeks’ gestation, the use of 2% chlorhexidine for skin asepsis before invasive procedures, and a change of dressings used for Broviac lines (see Figure 2). This was a significant change in neonatal practice; therefore walk-arounds on the unit and staff education sessions were arranged to understand the perceived barriers before the introduction of these IPC changes.

Taken together, these measures resulted in a significant reduction in MRSA infections (see Figure 1). The implementation of weekly screening initially resulted in a spike in colonization rates. However, through the combination of the above interventions, MRSA bacteremia have been eradicated within the Leeds neonatal service, with no cases of MRSA bacteremia since 2009. While we propose that our IPC interventions led to the reduction in neonatal MRSA infection and colonization rates, we recognize the potential impact of extrinsic factors, such as pathogen variation, wider antibiotic use, community transmission, environmental conditions, and improved neonatal nutrition may affect these outcomes.

## MSSA bacteremia in the Leeds neonatal service

Despite MRSA control, MSSA remained a challenge in the Leeds neonatal service. Analysis of MSSA bacteremia data demonstrated a significant portion of these cases occurred in babies born at less than 28 weeks’ gestation. Interestingly, Octenisan washes for MRSA prevention targeted babies of 28 weeks’ gestation or older, as younger babies were excluded due to concerns regarding skin integrity and thermostability.

**Figure 3.**
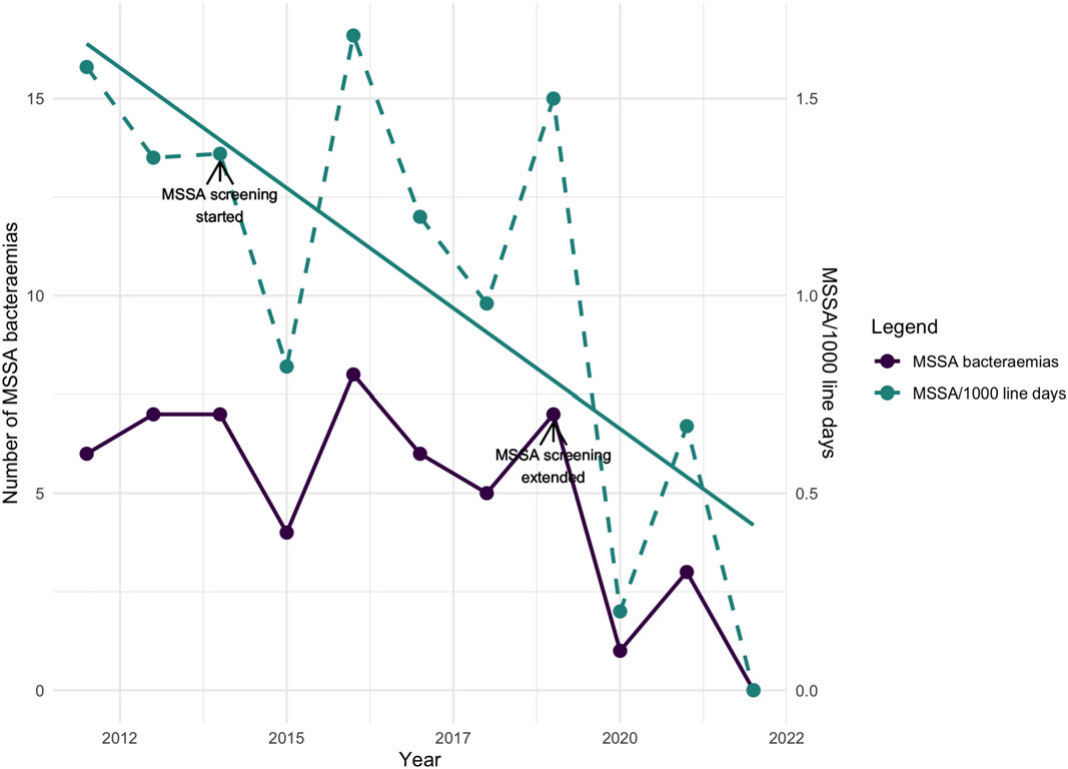
Number of methicillin-sensitive *Staphylococcus aureus* (MSSA) bacteremia and MSSA/1,000 central line days. Arrows signify the year in which interventions were implemented and extended.

In 2014, a screening program for MSSA colonization in babies less than 28 weeks’ gestation was implemented. This included admission and weekly screening swabs until 28 weeks postconceptual age. At this time, the standard daily decolonization with Octenisan began. Upon a positive MSSA screening swab, the baby received decolonization. This intervention significantly reduced MSSA in this population as shown in Figure 3.

Despite this initial screening intervention, MSSA bacteremia persisted in infants born before 28 weeks but occurred after they reached 28 weeks postconceptual age. To address this, in 2019, weekly screening was expanded to all such infants for their entire admission, leading to a further reduction of MSSA bacteremia rates (Figure 3). Additional enhancements were also introduced in 2019, including increased scrutiny of aseptic practices, the implementation of Matching Michigan checklists for line insertions, an individual patient infection prevention booklet mandating daily line dressing assessments, with changes only necessitated for soiled or lifting dressings, and revised humidity and skin care protocol.

## Staffing levels and their impact on MSSA rates

Understaffing results in decreased compliance with IPC measures.^26^ For neonatal care, nursing staff levels are based on care requirements and described by BAPM;^25^ high dependency care requires at least a 1:3 ratio, and intensive care necessitates a minimum 1:1 ratio. A retrospective analysis of the Leeds neonatal service from March 2008 to December 2011 showed a notable negative correlation between staffing levels and monthly *S. aureus* rates, with diminished staffing associated with higher *S. aureus* culture rates (Spearman’s correlation coefficient = −0.316; *P* = .03, unpublished data). Those months with lower staffing compliance (<80%) were associated with higher rates of *S. aureus* isolation. This implies that reduced staffing might increase the prevalence of *S. aureus* in the NICU, even with other IPC measures in place. The exact reasons behind this association may be complex and influenced by various factors such as length of stay and high-risk interventions. Nonetheless, the findings underscore the potential impact of staffing levels on infection rates in the NICU setting.

## Conclusion

The profound ramifications of MRSA infections, particularly within neonatal populations, underscore the urgent need for evidence-based interventions. Though MRSA control strategies adapted from adult populations have shown promise, they are not universally applicable, as indicated by the distinct challenges faced within the neonatal context. Robust evidence examining the efficacy of decolonization strategies and their potential longer-term effects on the neonatal microbiome are needed. Additionally, knowledge gaps remain including the mechanisms of transmission of *S. aureus* within NICUs, including factors associated with parental seeding of the microbiome, and the possible effect of infant feeding practices. Furthermore, while some risk factors for *S. aureus* colonization are known, there is a need for comprehensive identification and quantification of these factors in neonates to better aid clinicians in risk stratification. Table 2 summarizes the pivotal points concerning MRSA/MSSA interventions and outcomes.

**Table 2.** Key points and summary. Methicillin-resistant *Staphylococcus aureus* (MRSA)

Topic	Key points from the literature
MRSA prevention in NICU	Decisions based on local epidemiology, variation in practices, need for evidence to guide neonatal practice
Decolonization procedures	Very varied, 73.8% units routinely decolonized MRSA-colonized infants with >15 different regimens
Universal decolonization	More effective in adult ICU than targeted decolonization resulting in a 37% reduction in MRSA-positive cultures and a 44% reduction in bloodstream infections from any pathogen. The approach we have used in a limited way (Octenisan only, not Mupirocin)
Guidance NICU surveillance	No universal guidelines for surveillance and management CDC recommends sampling at least the anterior nares in NICU patients is recommended
Unintended consequences of decolonization	Potential negative impact on the neonatal nasal microbiome and unresolved optimal agent

Topic	Key interventions used within the Leeds neonatal service
MRSA/MSSA intervention: Staff	Increased staffing ratios, universal compliance with aseptic training. Staff MRSA screening and decolonization. Weekly interdisciplinary infection control meeting
MRSA/MSSA intervention: Environment	Increase cot spacing, de-cluttering, and refurbishment. Improved access to PPE, comprehensive cleaning audit
MRSA/MSSA intervention: Patient	Daily Octenisan washes, screening for MSSA skin colonization, new dressing packs. Individual IPC records. Skin preparation with 2%chlorhex (0.5% pre-LP), universal frequent IPC training and assessment

Note. ICU, intensive care unit; CDC, Centers for Disease Control and Prevention; PPE, personal protective equipment; MSSA, methicillin-sensitive *Staphylococcus aureus*; IPC, infection prevention and control; LP, lumbar puncture.

While MRSA has been the target of most NICU *S. aureus* prevention and control programs, MSSA may cause comparable morbidity and mortality and is likely more prevalent in most units.^19^ Ericson and colleagues recently reported that MSSA was responsible for 2.5 times more infections than MRSA.^18^ Shane and colleagues’ study of 8,444 very low birth weight neonates with *S. aureus* bacteremia or meningitis demonstrated that MSSA was nearly thrice as prevalent as MRSA and both organisms were associated with high mortality.^27^ A higher absolute burden of disease and mortality from MSSA compared with MRSA justifies refocusing prevention strategies to include MSSA in addition to MRSA.

The experience of the Leeds neonatal service emphasizes the importance of an adaptive, multifaceted approach to managing MRSA and MSSA colonization and infections. Increasing staffing levels and physical refurbishment of the unit posed significant challenges, primarily due to funding and the logistical constraints imposed by bed closures. Despite these hurdles, staff enthusiasm and engagement in educational and empowerment initiatives were notable. The decision-making process surrounding the screening strategy emerged as particularly complex, hindered by the scarcity of available data. Identifying the most impactful intervention is difficult; however, the enhancement of communication, staff engagement, and weekly screening with targeted decolonization were key contributors to success.
